# Editorial: Zebrafish as a tool for neurosciences: evolutionary conservation and translational relevance

**DOI:** 10.3389/fnbeh.2023.1238952

**Published:** 2023-06-28

**Authors:** José G. Ortiz, Robert Gerlai

**Affiliations:** ^1^School of Medicine, University of Puerto Rico, San Juan, Puerto Rico; ^2^Department of Psychology, University of Toronto Mississauga, Mississauga, ON, Canada; ^3^Department of Cell and System Biology, University of Toronto, Toronto, ON, Canada

**Keywords:** zebrafish, biomedical research, translational relevance, evolutionary conservation, behavioral neuroscience

Life on Earth started just over 3.5 billion years ago. The last common ancestor of current-day fish and mammals lived about 0.4 billion years ago. That is, the first 3.1 billion years of biological evolution are shared between these two vertebrate taxa. The result of this long common evolutionary history is that there are a large number of evolutionarily conserved features between fish and even our species, humans. Evolutionary biologists agree that current-day fish do not dramatically differ from their 400-million-year-old ancestors. Modern fish remained relatively simple compared to other vertebrates of today. On the other hand, mammals do not resemble fish and have undergone substantial evolutionary changes, e.g., from the neuroscientist's perspective, have become a lot more complex during the past 400 million years. As an example, consider that the zebrafish brain contains about four orders of magnitude fewer neurons than the human brain. This simplicity, coupled with the more ancient “design” of modern fish, is the reason why many zebrafish scientists argue for using this small and easy-to-keep animal in the laboratory for biomedical research. The zebrafish is an excellent translational tool through which evolutionarily ancient, and thus fundamentally important, mechanisms of complex mammalian (and human) biological phenomena may be studied.

This Research Topic brings together a variety of zebrafish studies exemplifying the above points. For example, Jacobs and Ryu discuss how zebrafish larvae may be employed to tackle a vexing problem in neuropsychology research and the human clinic: individual variability.

Bellot et al. also explore the role of individual differences, namely along the bold vs. shy axis, in acute alcohol rewarded place preference in zebrafish. They argue that translational studies using zebrafish will help identify factors that predispose humans to compulsive drinking. Blasiak et al. focus on relaxins (a family of peptide hormone neuromodulators) and discuss the evolutionary conservation of gene expression, ligand-receptor interaction and protein function, and the translational relevance of zebrafish in the analysis of human neuropsychiatric diseases.

Tran and Prober show how the zebrafish can be employed in the analysis of mechanisms underlying sleep and for the modeling of human sleep disorders. They discuss methods, including CRISPR/Cas system-generated mutants and their screening, for the identification of the genetic bases of human sleep disorders.

Fasano et al. explain how one can utilize the power of the comparative approach (comparison of distinct species, e.g., the zebrafish and human) in the context of brain development and its disorders. They review whole-brain/organism-based research as well as *in vitro* neuronal ensemble analyses that may allow the discovery of the mechanisms/genetics of pathological processes associated with human neurodevelopmental disorders.

Giffen et al. utilize the differences between mammals and the zebrafish, namely, the ability of the latter to regenerate hair cells. They identify uniquely expressed orthologous gene candidates involved in the proliferation and differentiation of non-sensory supporting cells to hair cells in the zebrafish inner ear in the hope to uncover targets for hair cell regeneration in mammals.

The above studies and reviews, along with the rapidly growing literature on zebrafish neuroscience, should persuade the reader of the utility of zebrafish in translational, biomedical, research. This collection emphasizes one key message, common to these studies: that comparative analysis is a powerful way of establishing translational relevance. For example, if one compares the results obtained in a mouse study with those gathered from the analysis of the zebrafish, one may be able to identify overlapping mechanisms, genes, protein targets, and biochemical mechanisms that are common to the two species. These evolutionarily conserved features will likely translate well to our species, and thus will significantly advance understanding of human biology, including the diseases of our brain.

## Author contributions

All authors listed have made a substantial, direct, and intellectual contribution to the work and approved it for publication.

